# Central serous chorioretinopathy and systemic corticosteroids in rheumatic diseases: report of three cases

**DOI:** 10.1186/s12891-015-0843-4

**Published:** 2015-12-05

**Authors:** Elia Valls Pascual, Lucía Martínez-Costa, Fernando Santander

**Affiliations:** Rheumatology Department, Hospital Universitari Doctor Peset, Juan de Garay, 21, 46017 Valencia, Spain; Ophthalmology Department, Hospital Universitari Doctor Peset, Valencia, Spain

**Keywords:** Central serous chorioretinopathy, Corticosteroids, Rheumatic diseases

## Abstract

**Background:**

Central serous chorioretinopathy is a disorder often related to systemic corticosteroids, drugs commonly used in rheumatologists’ clinical practice. Central serous chorioretinopathy prognosis is generally good but in some cases, it may lead to substantial loss of vision resulting in an important functional limitation for patients.

It is very important to distinguish this pathology from other diseases involving retinal detachment. When central serous chorioretinopathy and uveitis coexist, it is mandatory to distinguish serous retinal detachment from a uveitis worsening, as the respective treatments can be radically different.

**Case presentation:**

We describe three cases of central serous chorioretinopathy in patients taking systemic corticosteroids due to rheumatological diseases (ankylosing spondylitis, systemic lupus erythematosus and Behçet’s disease). They were diagnosed and managed at our Multidisciplinary (Rheumatology-Ophthalmology) Uveitis Clinic. All three cases improved after corticosteroids dose tapering.

**Conclusion:**

Central serous chorioretinopathy must be kept in mind by rheumatologists as it is related to systemic corticosteroids.

## Background

Central serous chorioretinopathy (CSCR) is a disease characterized by serous detachment of the neurosensory retina and/or the retinal pigment epithelium (RPE). The most accepted physiopathologic mechanism is the leakage of vascular fluid through a focal leak in the RPE into the subretinal space as a consequence of choroidal vessel hyperpermeability.

When the macula is affected, CSCR results in blurred vision, metamorphopsia and micropsia.

CSCR predominantly affects middle-aged adults. Several risk factors have been described, the most recognized ones being: type-A personality and psychological stress [1, 2], pregnancy [3], and increased levels of corticosteroids of exogenous and endogenous origin [4–10].

Although CSCR prognosis is generally good, this illness often affects the macula leading to a loss of visual acuity (VA) and resulting in an important functional limitation for patients.

Furthermore, when not diagnosed and treated correctly, CSCR can become chronic leading to diffuse damage of the RPE, permanent loss of VA, and complications involving choroidal neovascularization.

Herein we describe three cases of CSCR diagnosed and managed at our Uveitis Clinic.

## Case presentation

### Case 1

A 45-year-old man diagnosed with HLA B27 positive ankylosing spondylitis. During the follow up in our department, the patient showed axial and peripheral articular involvement and suffered several episodes of anterior acute uveitis in the right eye. He was treated with NSAIDs and methotrexate 15 mg/week p.o.

In December 2012 the patient developed a new acute anterior uveitis episode on the right eye, which was diagnosed and initially managed in another medical center. He was treated with prednisone 60 mg/day after topical and periocular corticosteroid injection failure. While on this treatment, the patient was referred to our consulting room. We found no inflammatory signs on the anterior chamber by ophthalmological examination (uveitis episode had been resolved) but the patient kept complaining about blurred vision. Funduscopic examination showed a retinal detachment which was confirmed and characterized as CSCR by optical coherence tomography (OCT).

Because of the absence of inflammatory signs and the possible association of CSCR with systemic corticotherapy, we decided to decrease the prednisone dose until full discontinuation leading to resolution of the retinal detachment.

Four months after prednisone discontinuation the patient developed a new uveitis episode which was treated with periocular triamcinolone injection, to which adalimumab 40 mg EOW was added due to the aggressive course even under methotrexate treatment. With this combined treatment, the patient has not had any recurrences until the present day, and treatment with systemic corticosteroids has not been necessary.

### Case 2

A 37-year-old woman with a history of sigma adenocarcinoma treated with surgery. She was diagnosed with systemic lupus erythematosus (SLE) with hematological, renal, articular and serous involvement. The following treatment was initiated: methotrexate 5 mg/week P.O. and prednisone 7.5 mg/day, as well as rituximab 375 mg/m2 104 weekly × 4 (off label use) only upon relapse. Previously she had been treated with chloroquine, but it had to be suspended as the patient developed an antimalarial related maculopathy.

**Fig. 1:**
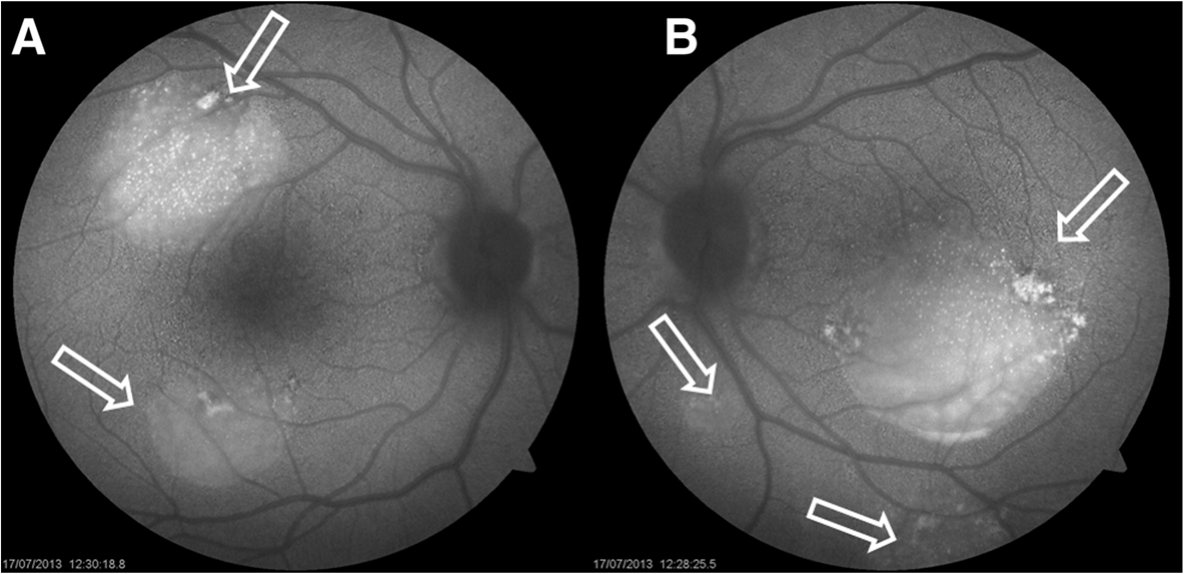
Autofluorescence images of case 2. **a**, **b** Multifocal central serous choroidopathy is shown in both eyes (arrows)

**Fig. 2:**
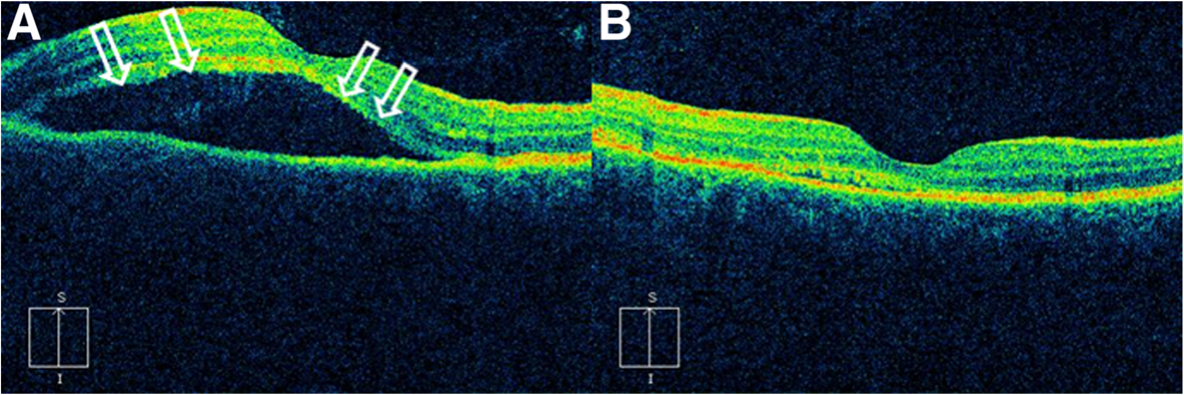
Left-eye optical coherence tomography images of case 2. **a** Serous retinal detachment (arrows). **b** Resolution of fluid collection after discontinuation of oral steroids

In June 2013 the patient was referred to our consulting office because of blurred vision of new onset. The previous month she had suffered an SLE relapse (pleuritis and deterioration of renal function in a context of lupus glomerulonephritis class III) that was treated with a new cycle of rituximab and prednisone 60 mg/day. On funduscopic examination, a retinal detachment was observed which was confirmed by autofluorescence and OCT (see Figs. 1 and 2).

A differential diagnosis between serous retinal detachment (SRD) related to SLE [11, 12] and corticosteroid-related CSCR was considered. We decided that the corticosteroid-related CSCR option was more reasonable because of an absence of ocular inflammatory changes and the good response to treatment of the previous SLE relapse, as well as finding no other clinical or analitical signs of SLE activity. Thus, we initiated a prednisone dose tapering until 5 mg/day, which led to the CSCR resolution.

Three months later the patient suffered a new relapse of SLE (pleuritis), so a new rituximab cycle was administered, thereby avoiding systemic corticosteroids except for a single dose of endovenous metilprednisolone 100 mg as rituximab premedication. The SLE relapse was resolved and the patient hasn’t had new ocular involvement until the present day.

### Case 3

A 45-year-old man with a history of recurrent pancreatitis of enolic etiology. He was diagnosed with Behçet’s disease with both mucous (oral and genital ulcers) and ocular involvement (bilateral papillitis and cystoid macular edema). The following treatment was initiated: cyclosporine 200 mg/day P.O. and prednisone (doses ranging from 5 mg/day to 60 mg/day depending on disease activity), as well as rituximab 1000 mg EOW × 2 (off label use) only upon relapse. Previously he had been treated with infliximab but had to be discontinued due to an infusion reaction.

In April 2012, the patient was referred to our consulting room because of blurred vision.

Funduscopic examination and OCT revealed bilateral vitritis as well as SRD. The patient was on treatment with cyclosporine 200 mg/day P.O. and prednisone 5 mg/day. As inflammatory signs were detected in addition to the SRD, we decided to add a new cycle of rituximab. This treatment led to vitritis resolution but SRD persisted on both eyes. At this point, we considered that the SRD could be related to chronic use of systemic corticosteroids, so we decided to decrease the prednisone dose to 2.5 mg EOD, not adding any other immunosuppressant drug.

Initially, SRD was resolved, but seven months later he suffered a new episode of uveitis on the left eye (papilitis, vitritis, and cystoid macular edema). A subtenon injection of triamcinolone 40 mg and a new cycle of rituximab were initiated. Systemic corticosteroids were avoided, with exception of an endovenous metilprednisolone 100 mg premedication prior to rituximab infusion. Following this latest treatment, inflammatory signs disappeared and no SRD has been detected until the present day.

## Discussion

CSCR is a disease that must be kept in mind by rheumatologists, as it is a relatively frequent disorder [13] related to systemic corticosteroids, drugs commonly used in our clinical practice.

It is also important to have a fluid communication with ophthalmologists, who must do a good differential diagnosis with other diseases associated with retinal detachment such as: Vogt Koyanagi Harada, myopia, diabetes mellitus and central retinal vein thrombosis, as well as diseases that can have a similar course of action such as chronic CSCR (which like age-related macular degeneration, should be suspected in elder patients). This is very important, as treatment of these diseases can be radically different: the initial treatment for corticosteroid related CSCR should be corticoids withdrawal, what would worsen the course of inflammatory chorioretinopathies.

In the event of CSCR and uveitis coexistence, as in our first and third cases, it is mandatory to distinguish serous retinal detachment from uveitis worsening, as the symptomatology can be very similar [14]. For this reason, an optimal ophthalmological examination is necessary, as well as the use of additional diagnostic tools such as OCT, autofluorescence and fluorescein angiography [15]. When CSCR is diagnosed in a context of previous or concomitant uveitis, immunosuppressants other than corticosteroids should be initiated, or previous immunosuppressants doses increased. When corticosteroids cannot be avoided because of emergency or limited access to other drugs, topical or periocular forms should be employed.

A limitation of our approach to these cases is that we didn’t consider other risk factors for CSCR development. Nonetheless, as systemic corticosteroids use is the better one described, we considered this risk factor as the most probably related to CSCR in our cases. Another possible limitation of this report is the absence of additional studies including fluorescein and ICG angiography as well as some examination technologies (adaptive optics, electroretinography, microperimetry, or contrast sensitivity testing) for the diagnosis and especially for the patients’ follow-up, as it is described that these technologies can detect subclinical damage [15].

## Conclusions

CSCR must be kept in mind by rheumatologists as it is related to systemic corticosteroids, drugs commonly used in our daily practice.

In the event of CSCR and uveitis coexistence, it is mandatory to distinguish serous retinal detachment from uveitis worsening.

## Consent

Written informed consent was obtained from the patients for publication of this Case report and any accompanying images. A copy of the written consent is available for review by the Editor of this journal.

## Ethical board review statement

This material has not been published and is not under consideration elsewhere. This study receives no financial support.

## Abbreviations

CSCR: Central serous chorioretinopathy
OCT: Optical coherence tomography
RPE: Retinal pigment epithelium
SLE: Systemic lupus erythematosus
SRD: Serous retinal detachment
VA: Visual acuity
